# A systematic review and meta-analysis of the prevalence of bipolar disorder among homeless people

**DOI:** 10.1186/s12889-020-08819-x

**Published:** 2020-06-09

**Authors:** Getinet Ayano, Shegaye Shumet, Getachew Tesfaw, Light Tsegay

**Affiliations:** 1 Research and Training Department, Amanuel Mental Specialized Hospital, Addis Ababa, Ethiopia; 2 School of Public Health, Curtin University, Perth, WA Australia; 3 Department of Psychiatry, College of Medicine and Health Sciences, University of Gondar, Gondar, Ethiopia; 4 Department of Psychiatric Nursing, College of Health Sciences, Axum University, Axum, Ethiopia

**Keywords:** Prevalence, Bipolar disorder, Homeless, Systematic review, Meta-analysis

## Abstract

**Background:**

Bipolar disorder (BD) is a common severe mental disorder among homeless people and is associated with an increased risk of disability and mortality from suicide, medical causes (including HIV/AIDS, hepatitis infection, hypertension, and tuberculosis), as well as substance use disorders. However, a systematic synthesis of the existing evidence on the subject is lacking. To fill this gap in the literature, this study aimed to carry out systematic review and meta-analysis to determine the consolidated prevalence of BD among homeless people.

**Methods:**

In this systematic review and meta-analysis, we searched Embase, PubMed, and Scopus to identify pertinent studies that reported the prevalence of BD among homeless people in March 2019. Random effect meta-analysis was employed to pool data from the eligible studies. Subgroup and sensitivity analysis was conducted and Cochran’s Q- and the I^2^ test were utilized to quantify heterogeneity. Publication bias was assessed by using Egger’s test and visual inspection of the symmetry in funnel plots.

**Results:**

Of 3236 studies identified, 10 studies with 4300 homeless individuals were included in the final analysis. Among the 10 studies, five studies used the Diagnostic Statistical Manual of Mental disorders (DSM), three studies used Mini-International Neuropsychiatric Interview (MINI), one study used Schedule for Clinical Assessment of Neuropsychiatry (SCAN), and one study used Composite International Neuropsychiatric Interview (CIDI) to assess BD among homeless individuals. Based on the results of the random effect model, the prevalence of BD among homeless people was 11.4% (95% CI; 7.5–16.9). The prevalence of BD was 10.0% (95% CI; 3.1–27.9) in Europe and it was 13.2% (95% CI; 8.9–19.3) in other countries. Moreover, the prevalence of BD was 11.5% (95% CI; 5.5–22.3) for studies that used DSM to assess BD and it was 11.0% (95% CI; 6.1–19.2) for studies that used other instruments (MINI, SCAN, and CIDI).

**Conclusion:**

Our meta-analysis demonstrated that BD is highly prevalent among homeless individuals, underlying the importance of early screening and targeted interventions for BD among homeless individuals.

## Background

According to a report from Yale University, around 150 million people, or roughly 2% of the global population were classified as homeless, and as many as 1.6 billion people (20% of the world population) lacked adequate housing [1]. Among the many causes of homelessness— mental illness, sexual assault, poverty, domestic violence, unemployment, addictions, a critical shortage of affordable housing, social isolations, family breakdown, adverse childhood experiences, and financial difficulties were reported as the most common factors in different studies [2–6].

Numerous studies have reported a higher prevalence of psychiatric disorders among homeless people. The diagnosis of psychiatric disorders among homeless people ranged from 25 to 50% depending on the studies [7–10]. The prevalence rates are reported as high as 92% among those who are street homeless [11]. The diagnoses of psychiatric disorders among homeless people associated with an elevated risk of mortality from suicide and general medical [12–15] and drug-related causes [16].

Bipolar disorder (BD) is one of the most common mental disorders among homeless people that is associated with an increased risk of disability, substance/drug abuse as well as mortality from suicide, and medical causes (including HIV/AIDS, hepatitis infection, hypertension, and tuberculosis), as well as substance use disorders [12–14, 17, 18]. Bipolar disorder is estimated to affect 2.41 to 42.42% of the homeless people depending on the studies [17–22]. The consequences of BD among homeless people may be severe and far-reaching; it negatively affects the person experiencing BD as well as their family [12, 16, 23, 24].

Even though, the existing epidemiologic evidence show a greater burden of BD among homeless people coupled with its negative consequence associated with a greater risk of mortality, higher medical comorbidity, increased substance use, risky behaviors, disability and poor quality of life for the affected people, to date there are no systematic review and meta-analysis that reported the pooled prevalence of BD among homeless people. Thus, the objective of this systematic review and meta-analysis is to estimate the prevalence of BD among the homeless by pooling the existing data on the prevalence of BD among the homeless population and provide a recommendation for future research and clinical practice.

## Methods

### Research design and method

The PRISMA (Preferred Reporting Items for Systematic Reviews and Meta-Analyses) guidelines [25] was used to conduct this systematic review and meta-analysis. We used a predesigned protocol for searching, data abstraction, inclusion-exclusion criteria, quality evaluation, as well as data synthesis and analysis.

### Data source and selection process

An extensive search of three electronic databases (Embase, PubMed, and Scopus) was conducted in March 2019. We employed our search in PubMed using the following terms and keywords: (((“bipolar disorder”[MeSH Terms] OR (“bipolar”[All Fields] AND “disorder”[All Fields]) OR “bipolar disorder”[All Fields]) OR (“mental disorders”[MeSH Terms] OR (“mental”[All Fields] AND “disorders”[All Fields]) OR “mental disorders”[All Fields] OR (“mental”[All Fields] AND “illness”[All Fields]) OR “mental illness”[All Fields]) OR (“mental disorders”[MeSH Terms] OR (“mental”[All Fields] AND “disorders”[All Fields]) OR “mental disorders”[All Fields] OR (“psychiatric”[All Fields] AND “disorder”[All Fields]) OR “psychiatric disorder”[All Fields])) AND ((“epidemiology”[Subheading] OR “epidemiology”[All Fields] OR “prevalence”[All Fields] OR “prevalence”[MeSH Terms]) OR magnitude [All Fields] OR (“epidemiology”[Subheading] OR “epidemiology”[All Fields] OR “epidemiology”[MeSH Terms]))) AND ((“homeless persons”[MeSH Terms] OR (“homeless”[All Fields] AND “persons”[All Fields]) OR “homeless persons”[All Fields] OR “homeless”[All Fields]) OR (“homeless persons”[MeSH Terms] OR (“homeless”[All Fields] AND “persons”[All Fields]) OR “homeless persons”[All Fields] OR “homelessness”[All Fields])). For Embase and Scopus database searching we applied specific-subjects headings suitable for each database. We also conducted a manual search of the reference lists of eligible articles to supplement the electronic database search.

### Inclusion and exclusion criteria

To be included in this systematic review and meta-analysis studies had to satisfy the following criteria: (1) observation study in design was used to conduct the study (cross-sectional, case-control or cohort studies); (2) the source population was homeless people (conducted among homeless people); (3) measured the prevalence of bipolar disorders or data to calculate the prevalence was reported; (4), were a primary study in type. Additionally, studies were not excluded based on the type of homelessness (studies involving any type of homelessness were included without restriction). Excluded were reviews, book reviews, commentaries, and conference presentations.

### Selection of studies for the inclusion in the systematic review and meta-analysis

The corresponding author (GA) identified studies and subsequently screened them using their titles as well as abstract against the inclusion and exclusion criteria. Full-text studies were selected for further valuation by the author. This author further evaluated the full text of each article and consequently retained those full-text articles to be included in the present systematic review and meta-analysis.

### Methods for data extraction and quality assessment

Two reviewers (SS and GT), independently conducted manual data extraction from the eligible articles. As suggested by PRISMA guidelines [25], we utilized predefined data extraction to abstract pertinent data from the eligible studies. The following information was extracted from all eligible full-text articles: the name of authors, tools used, location, study design and setting, a sample size, year of publication, and prevalence of BD. A modified version of the Newcastle-Ottawa Scale (NOS) was used to evaluate the quality of eligible articles [26].

### Data synthesis and analysis

The meta-analysis was conducted by using comprehensive meta-analysis software version 3. A random-effect meta-analysis was used to pool prevalence data from the included studies. Q statistic and I^2^ statistics were employed to evaluate the heterogeneity [27]. The magnitude of heterogeneity between studies was evaluated by I^2^statistics [27] and values of 25, 50 and 75% denoted low, medium and high, respectively [28]. The instrument used to measure BD, the geographic location of the study, and the quality of the eligible articles was used as a moderator to quantify the possible source of heterogeneity between the studies. The evidence of publication bias was determined by using a funnel plot and Egger’s regression tests.

## Results

### Identification of studies

**Fig. 1:**
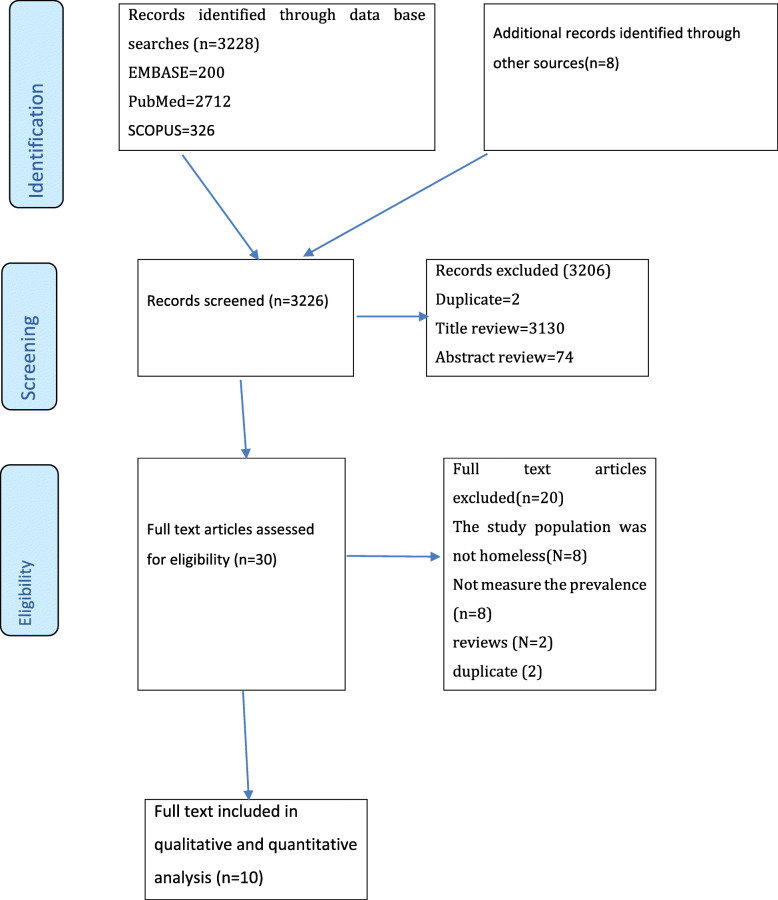
PRISMA flowchart of review search. This figure illustrates the process of searching for relevant studies from the three reputable databases including identification, selection, eligibility and inclusion of the studies depending on the predefined criteria

A total of 3236 eligible articles were identified by our electronic databases and manual searches. After title and abstract verification and eliminating duplicate articles, 30 articles remained for full-text screening. Of these, 20 were excluded, as they did not meet the inclusion criteria. The major reasons for the exclusion include: (1) the study population was not on homeless (*n* = 8), (2) not measured prevalence of bipolar disorders (n = 8), (3) reviews (*n* = 2), and (4) duplicate (n = 2). Thus, a full-text of 10 studies, which satisfied the eligibility criteria, were included in the final analysis (Fig. 1, supplementary files 1 and 2).

### Characteristics of included studies

Table 1 summarizes the characteristics of the included studies. A total of 10 studies were included in the final systematic review and meta-analysis. The included articles were performed in five countries representing a total of 4300 homeless people. From the total, three studies were conducted in Canada [31–33], two studies were conducted in the USA [29, 30], two studies in Germany [20, 21], one in Ireland [33], one in Brazil [35], and one was conducted in France [22]. The included studies were published between 2001 and 2017, with the sample size ranging between 38 participants in Ireland and 2088 participants in Canada. Among the 10 studies, five studies used the Diagnostic Statistical Manual of Mental disorders (DSM), three studies used Mini-International Neuropsychiatric Interview (MINI), one study used Schedule for Clinical Assessment of Neuropsychiatry (SCAN), and one study used Composite International Neuropsychiatric Interview (CIDI) to assess BD among homeless individuals.

**Table 1:** Distribution of studies on bipolar disorder among homeless people based on study year name, year of publication, sample size, instrument, country, and prevalence

Author (year) (reference number)	Sample size	Tool	Country	Type of homelessness	Prevalence
Koegel et.al. 1988 [29]	328	DSM	USA		10.6% (*n* = 35)
Fichter et.al. 2001 [20]	265	DSM	Germany	Shelter users, service users and street dwellers	8.3% (*n* = 22)
Connolly et.al. 2008 [30]	60	DSM	USA		5% (n = 3)
Noel et.al. 2016 [31]	497	DSM	Canada		*N* = 19% (*N* = 97)
Topolovec-Vranic et al. 2017 [32]	2088	MINI)	Canada		12.6%(*N* = 263)
Prinsloo et.al. 2012 [33]	38	DSM	Ireland		5.3%(n = 2)
Kovess et.al. 1999 [22]	715	CIDI	France		3.6%(n = 26)
Greifenhagen et.al. 1997 [21]	32	DSM	Germen		44% (*n* = 14)
Strehlua et.al. 2012 [34]	193	MINI	Canada		28% (*n* = 53)
Heckert et.al. 1999 [35]	83	SCAN	Brazil		2.41% (n = 2)

### Quality of the included studies

The quality of the eligible studies was evaluated by using the NOS with modification. Among the total, five articles found to be high quality (NOS score 8 and above), three were low quality (NOS score less than or equal to 5), and two were moderate quality studies (NOS score between 6 and 7 inclusive). The results show that most of the included studies had no significant risk of bias in the selection of the participants, as well as ascertainment of the outcome. Also, our sensitivity and meta-regression analysis revealed that the quality of the studies had no significant impacts on the over prevalence estimates of BD among homeless individuals (Supplementary file 3).

### The prevalence of BD among homeless people (meta-analysis)

**Fig. 2:**
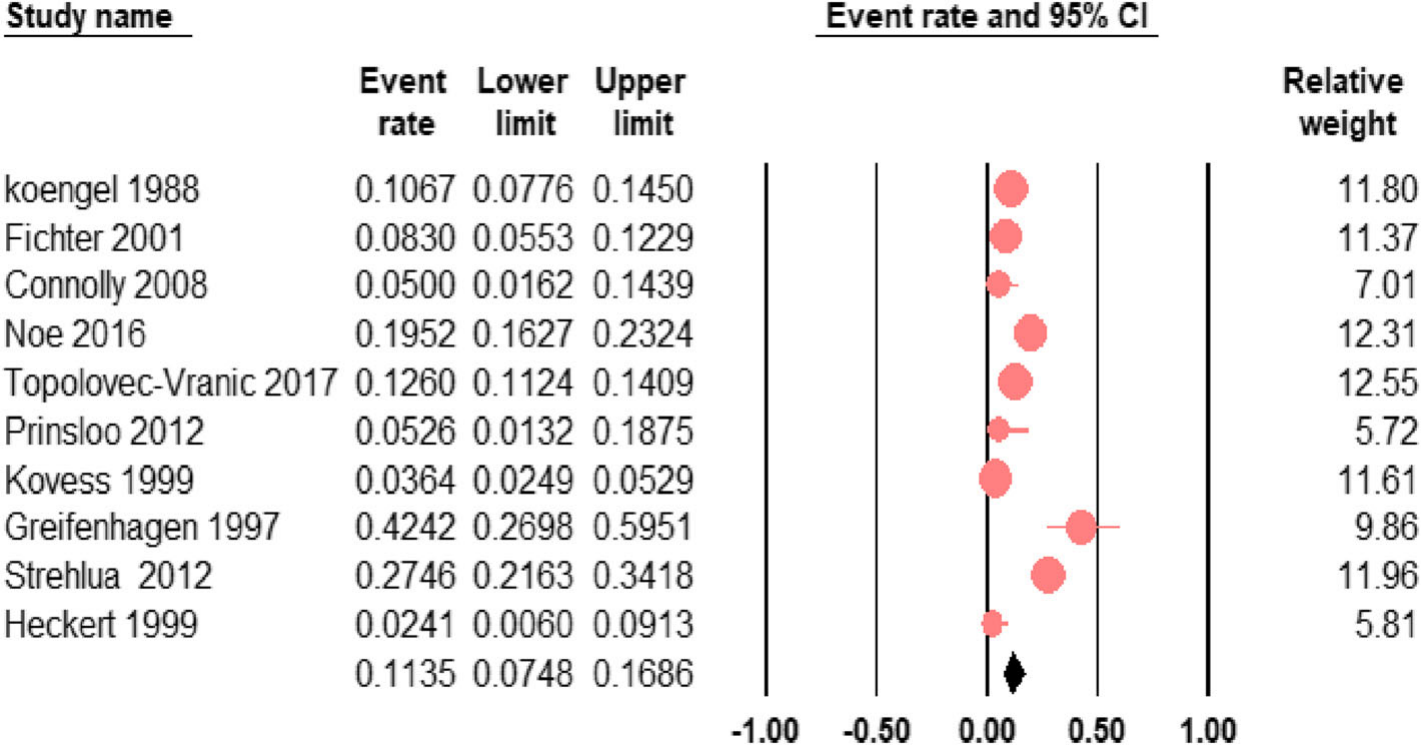
forest plot of the prevalence of bipolar disorder among homeless people. The figure shows the results of the meta-analysis of the studies on bipolar disorder among homeless people using random-effect model

The pooled prevalence estimate of BD among homeless people was 11.4% (95% CI; 7.5–16.9) with significant heterogeneity between the studies (*I*^2^ = 93.9%; *p* < 0.001). This pooled prevalence of BD among homeless people was yielded based on all 10 included studies and is illustrated by the forest plot in Fig. 2. The reported pooled prevalence was based on the random-effects model that we employed to account for the existing heterogeneity.

### Subgroup analysis

#### The prevalence of BD among homeless people by country (continent)

In this systematic review and meta-analysis, we have conducted a subgroup analysis by using the country/continent as a moderator (studies conducted in Europe vs. other countries). For this analysis, we included four studies from Europe [20–22, 33], three studies from Canada [31–33], two studies from the USA [29, 30], and one study from Brazil [35]. The prevalence of BD was 10.0% (95% CI; 3.1–27.9) in Europe and it was 13.2% (95% CI; 8.9–19.3) in other countries. Significant heterogeneity was found for both studies conducted in Europe (*I*^2^ = 94.5%, *p* < 0.0001) as well as studies conducted in other countries (*I*^2^ = 94.1%, *p* < 0.0001) (See Table 2).

**Table 2:** Subgroup and sensitivity analysis of the prevalence of bipolar disorder among homeless participants

Subgroup	Number of studies	Estimates		Heterogeneity across the studies		Heterogeneity between groups (*P*-value)
		Prevalence (%)	95% Confidence interval	I^**2**^ (%)	***P***-value	
**Country (continent)**						
Europe	4	10.0	3.1–27.9	94.5	< 00010.	0.635
Others	6	13.2	8.9–19.3	94.1	< 0001	
**Instrument used**						
DSM	5	11.5	5.5–22.3	87.0	< 0.0001	0.926
Other	5	11.0	6.1–19.2	96.1	< 0.0001	
**Quality of the study**						
High	5	9.9	6.2–15.3	94.4	< 0.0001	0.743
Moderate and low	5	11.8	4.3–28.6	88.4	< 0.0001	
**Sample size**						
400 and above	3	10.1	5.1–19.1	97.0	< 0001	0.791
Below 400	7	11.45	5.8–21.2	91.0	< 0001	

#### Subgroup analysis of the prevalence of BD among homeless people by the instrument used

We also conducted a stratified analysis by the type of instrument used to measure BD among the homeless people using a random effect model. The prevalence of BD was 11.5% (95% CI; 5.5–22.3) for studies that used DSM to assess BD and it was 11.0% (95% CI; 6.1–19.2) for studies that used other instruments (MINI, SCAN, and CIDI). The reported heterogeneity was significant for both studies conducted by DSM (*I*^2^ = 87.0%, p < 0.0001) and other instruments (*I*^2^ = 96.1%, *p* < 0.001) (See Table 2).

#### Subgroup analysis of the prevalence of BD among homeless people by the quality of studies

Furthermore, we also conducted a subgroup analysis by the quality of the eligible studies. The pooled prevalence of BD was found to be 9.9% (95% CI, 6.2–15.3) for high-quality studies and the pooled prevalence for both low and moderate-quality studies combined was 11.8%(95% CI, 4.3–28.6).

The reported heterogeneity was significant for high (*I*^2^ = 94.4; p < 0.001) as well as low and moderate (*I*^2^ = 88.4; p < 0.001) quality studies. (See Table 2).

#### Subgroup analysis based on the sample size of the study

The prevalence of BD was 10.1% (95% CI 5.1–19.1) for studies that included a sample of 400 and above homeless participants and it was 11.5% (95% CI; 5.8–21.2) for studies that included less than 400 homeless participants (See Table 2).

#### Sensitivity analysis

Sensitivity analysis was performed according to the country of study (origin of the study), the instrument used to measure BD, and the quality of the included studies to further explore the possible source of heterogeneity in the analysis of the prevalence of BD among homeless people. Our sensitivity analysis demonstrated that the prevalence of BD was slightly lower in Europe (10.0%) when compared with other countries (13.2%), although the variation was not statistically significant (*P* = 0.635) (see Table 2).

Moreover, we performed a sensitivity analysis based on the tools used to quantify BD among homeless people as a moderator. The findings demonstrated that the prevalence of BD among the homeless individuals was comparable between the studies conducted using the DSM and other instruments (see Table 2).

We also conducted a sensitivity analysis by using the quality of the included studies as a moderator. This analysis showed that the estimated prevalence of BD was found to be higher for low quality and moderate-quality studies (16.7%) as compared to high-quality studies (9.9%), although the difference was not statistically significant (*P* = 0.743) (see Table 2).

Furthermore, our sensitivity analysis revealed that the prevalence of BD among the homeless individuals did not significantly vary based on the sample size used to estimate the prevalence of BD among the participants (see Table 2).

Furthermore, to confirm whether the results of our final meta-analyses were heavily affected by study with greater relative weight, we performed a sensitivity analysis by removing a study with a higher relative weight [34] and the results revealed no significant variation in our final results. The prevalence was 10.0% (95% CI 6.5–15.1) after excluding a study with a larger relative weight [32].

#### Meta-regression

Firstly, we have performed a univariate regression analysis that directed the selection of the important variables to involve in the final meta-regression analysis. All independent variables with *P*-value < 0.8 were included in the final meta-regression model as suggested by Ferrari et.al. (Ferrari et al., 2013). In the final meta-regression analysis model, we quantified the impacts of sample size (400 and above and below 400), continent (country) (studies conducted in Europe and other countries), and, and the instrument used to measure depression (DSM and others). The overall proportion of variance explained by the above covariates in the final model was 8% (R^2^ = 0.08; *P* value = 0.772). All three independent variables such as diagnostic instruments, sample size, as well as a continent (country) were not significant determinants for the observed difference in the prevalence of bipolar disorders among homeless individuals (Table 3).

**Table 3:** Summary of the meta-regression analysis including sample size, country (continent where the study was conducted, and the quality of the studies in the model

Variables in the final model	Univariate model			Multivariable model		
	Coefficients	95% CI	*P*-value	Coefficients	95% CI	*P*-value
Sample size						
400 and above Below 400	Reference 0.188	Reference − 0.865-1.242	Reference 0.727	Reference −0.032	Reference −1.214-1.152	Reference 0.958
Country (continent)						
Europe Others	Reference 0.252	Reference −0.659-1.163	Reference 0.588	Reference 0.249	Reference −0.670-1.168	Reference 0.595
Quality of studies						
High Low/moderate	Reference 0.421	Reference −0.794-1.321	Reference 0.326	Reference 0.438	Reference −0.721-1.597	Reference 0.459

Test of the model (multivariable model): R^2^ = 0.08; *P*-value = 0.772

#### Publication bias

**Fig. 3:**
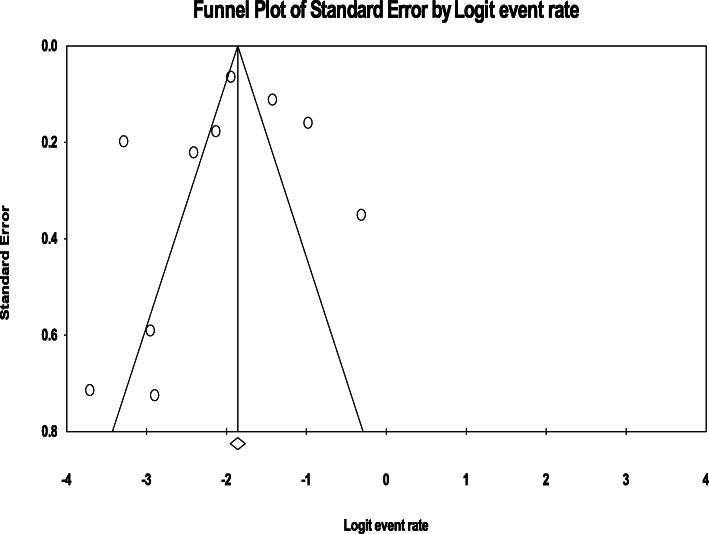
Publication bias for bipolar disorders among homeless people. Funnel plot of the risk of publication bias for the prevalence of BD among the homeless individuals

Figure 3 demonstrates the risk of publication bias. The analysis revealed that the funnel plot was symmetric and Egger’s regression tests provided no evidence of substantial publication bias for the prevalence of BD among the homeless people (B = -1.14, SE = 2.19, *P*-value = 0.792).

## Discussion

### Main findings

To the best of our knowledge, this is the first systematic review and meta-analysis that assessed the prevalence of BD among homeless people. Ten studies with a total of 4300 homeless people conducted across six countries were included in the final analysis. The findings of this systematic review indicated that the available epidemiologic evidence on the prevalence of BD among homeless people showed an apparent variation by the tools used to measure BD, the study location (the origin of the study), as well as the quality of the included articles, although the variation was not statistically significant. To measure BD, some of the articles employed screening instruments and some employed diagnostic instruments. Nine of the included studies were conducted in developed countries including 2 studies in the USA, 3 studies in Canada, and 4 studies in Europe and only one study was conducted in developing countries (Brazil).

This meta-analysis showed that the pooled prevalence of BD among homeless people was 11.4%. This result is remarkably higher (11.35-fold higher) than the reported prevalence of BD among the general population [36]. For example, a recent systematic review and meta-analysis conducted by Clement et al. found that the lifetime prevalence of bipolar disorders was found to be 1% [36], whereas, the reported prevalence ranges from 1 to 2.4% depending on the studies [36–39]. The possible reasons for the higher prevalence of BD among the homeless could be due to the greater prevalence rates of serious medical conditions including tuberculosis, HIV/AIDS and other medical conditions among the homeless people as compared to the reported prevalence in the general population [23, 40, 41], which in turn are associated with increased risks of bipolar and other psychiatric disorders in affected individuals [42–44]. The other possible reason for the observed variation in the prevalence of BD may be due to homeless people are more likely to experience traumatic events, such as physical and sexual abuse, which has been associated with the onset of a manic episode, earlier episodes, as well as early onset of bipolar illness in a risky group [45–51]. Another possible reason for a higher prevalence of BD among the homeless in the current study may be due to the remarkably greater prevalence of psychiatric and substance use disorders among homeless people such as depression, anxiety, psychosis, personality, and alcohol use disorders among homeless people as compared to the general populations [11, 29, 52, 53]. For example, a recent national-wide study conducted in Canada in 2018 found that 36.8% of homeless people with alcohol and substance use disorders (ASD) had comorbid BD [54]. Another study also showed that substance use disorders are common among homeless people with BD as compared to schizophrenia spectrum (SS) disorders and substance use problems including alcohol, cannabis, cocaine, and opiates were significantly linked with higher risks of bipolar disorders among the homeless study participants [55].

In our subgroup and sensitivity analysis, we found that the prevalence of BD was slightly lower in Europe (10.0%) when compared with other countries (13.2%), although the variation was not statistically significant (*P* = 0.635). We observed a wide variation in the prevalence of BD among homeless individuals ranging from 2.41% in Brazil to 44% in Germany. Additionally, among the three studies conducted in Canada, two of them reported a considerably higher prevalence of BD (above 20%) when compared with the pooled prevalence estimates (11.4%). There is a range of explanations for the observed differences in the prevalence of BD among homeless people across the countries. First, the methodologic difference including the instruments used to measure BD across the included studies is the possible reason for the observed remarkable variations. For example, in all the three studies conducted in Canada, BD among homeless people was measured by a screening tool (MINI) and studies conducted in other countries have used DSM, SCAN or CIDI. Second, the differences in the prevalence of the possible factors elevating the risk of BD among vulnerable groups such as physical, psychological, and sexual trauma’s, and other traumatic events and disasters, as well as serious medical conditions across the countries, are the other possible reasons for the observed variation in the prevalence of BD. Finally, the difference in the characteristics of the participants in the included studies could be the other possible reasons for the observed variation in the prevalence of BD across the countries. For instance, one of the studies conducted in Canada include homeless participants who had a history of traumatic brain injuries and in another study, the majority of homeless participants had comorbid other mental and substance use disorders [31–33], which possibly contributed to the observed high prevalence of BD in Canada. Our sensitivity analysis based on country (continent) revealed that the observed difference across the countries was not statistically significant (i.e. occurred by chance) (*P* = 0.635).

### Difference between the studies included in systematic review and meta-analysis

In the current review, we found significant heterogeneity between the studies which may be due to the location of the study (the origin of the study), the tools used to measure BD, the quality of the involved articles, and the study participants differed on numerous characteristics. However, our meta-regression analysis revealed that all three independent variables such as diagnostic instruments, sample size, as well as a continent (country) were not significant determinants for the observed difference in the prevalence of bipolar disorders among homeless individuals. We also found a variation in the prevalence of BD among the homeless people by the countries where the studies were conducted as well as the qualities of the included studies in the meta-analysis, although the observed variation was not statistically significant. To account for the observed heterogeneity across the studies, we have used a random effect meta-analysis where summary effect estimates are more conservative than fixed effect summaries in meta-analysis.

### Strength and limitations

The current review had several strengths: (1) the abstraction of data and quality assessment were conducted by two independent investigators to reduce the probable evaluator bias; (2) performing a subgroup, sensitivity, as well as meta-regression analysis depending on the origin of the study, the instruments used to estimate BD, as well as the quality of the studies, detect the possible risk of bias.

Several limitations of this systematic review and meta-analysis should be considered: First, in the present review, the vast majority of the studies included the final analysis were conducted in developed countries (90%, *n = 9*). Therefore, the reported prevalence of BD among homeless people may not represent the existing true prevalence of the disorder in developing countries. Second, a small number of studies were involved in our subgroup as well as sensitivity analysis, which may reduce the precision of the estimate. Third, we observed a remarkable heterogeneity across the studies. Fourth, the selection of studies by one reviewer (GA) is the other limitation of the study. This is because evidence suggests that using a second reviewer in the entire process can increase the number of studies to be included in the final reviews (reduce the probability of missing relevant studies) [56]. In fact, this is not a major concern in our meta-analysis since our publication bias analysis revealed no significant bias or small study effect (B = -1.14, SE = 2.19, *P*-value = 0.792), indicating the effects of missed studies (if any) were not significant.

### The implication of the findings for future research and clinical practice

The present systematic review has some implication for the future research and clinical practice; (1), the meta-analysis resulted a higher prevalence of BD among homeless people as compared to the reported prevalence in the general population, which strongly emphasize the need of further studies evaluating the possible contributing factors for this prevalence as well as better preventions and treatment strategies for this population group. (2), we found a few numbers of studies that estimated the prevalence of BD in developing countries. So, further studies are required to confirm and strengthen our findings. (3), most of the studies conducted in the past several years were focused on the mental health of adult homeless people, so that studies focusing on youth homeless people are strongly warranted.

## Conclusion

In conclusion, in this review, the pooled prevalence of BD among homeless people was remarkably high (11.4%). The prevalence of BD was 10.0% in Europe and it was 13.2% in other countries. Furthermore, the prevalence of BD was 11.5% for studies that used DSM to assess BD and it was 11.0% for studies that used other instruments (MINI, SCAN, and CIDI). High-quality studies aimed to determine the possible reasons for the higher prevalence of BD among homeless people as compared to the reported prevalence in the general population are warranted. Attention needs to be given for the mental health of homeless people and strengthening mental health services for homeless people such as the possible integration of mental health services for homeless with their medical care are recommended.

## Supplementary information

**Additional file 1: **Screenshot of document results from the three databases. This additional material shows snapshot of number of studies identified during the database search PubMed (*n* = 2712), Embase (*n* = 200), and Scopus (*n* = 316).

**Additional file 2: **Excluded full text studies with reasons of exclusion. The major reasons for the exclusion includes: (a) Not measured the prevalence of bipolar disorder (*n* = 8): (b) the study population was not homeless people (n = 8); (2) reviews (n = 2); (3) duplicate (n = 2).

**Additional file 3.** The Quality of the included studies based on the NOS quality score, a 9 point score, Score 7 and above represented good quality; 6 to 7 represented moderate quality; and 0 and 5 represented poor quality.

## Data Availability

All data generated or analyzed during this study are included in this article.

## Abbreviations

AIDS: Acquired Immune Deficiency Syndrome
CIDI: Composite International Neuropsychiatric Interview
DSM: Diagnostic Statistical Manual of Mental disorders
HIV: Human Immune Deficiency Virus
MINI: Mini International Neuropsychiatric Interview
NOS: Newcastle-Ottawa Scale
PRISMA: Preferred Reporting Items for Systematic Reviews and Meta-Analyses
SCAN: Schedule for Clinical Assessment of Neuropsychiatry
USA: United States of America
WHO: World Health Organization
