# Supplementary figures and images for: A systematic review and meta-analysis of the prevalence of bipolar disorder among homeless people
Source: BMC Public Health. 2020 Jun 9;20:731. doi: 10.1186/s12889-020-08819-x (PMC7282102; doi:10.1186/s12889-020-08819-x)

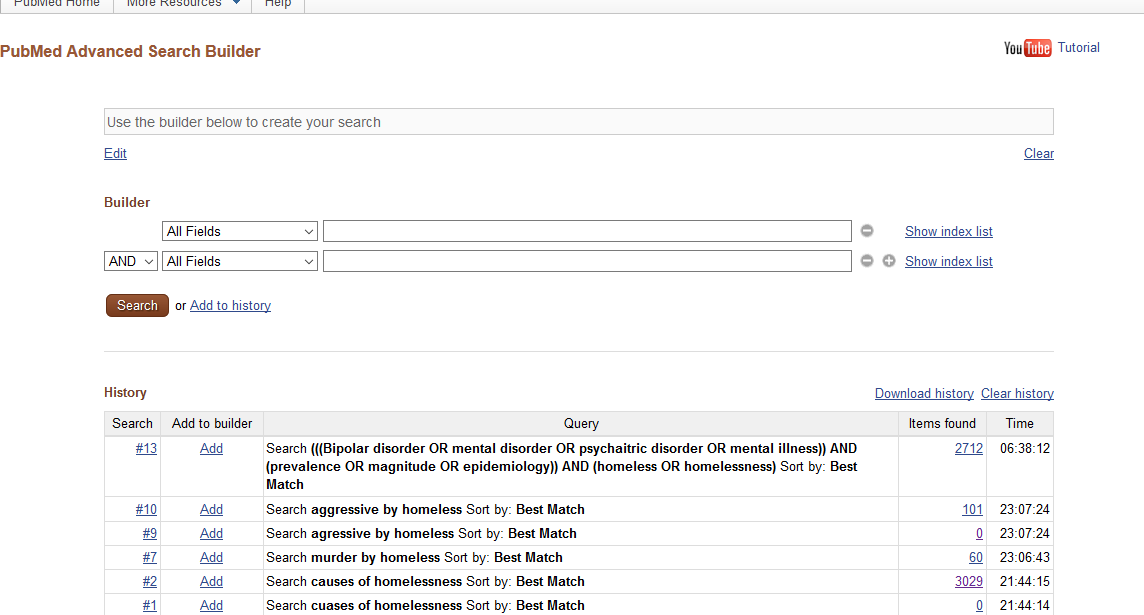


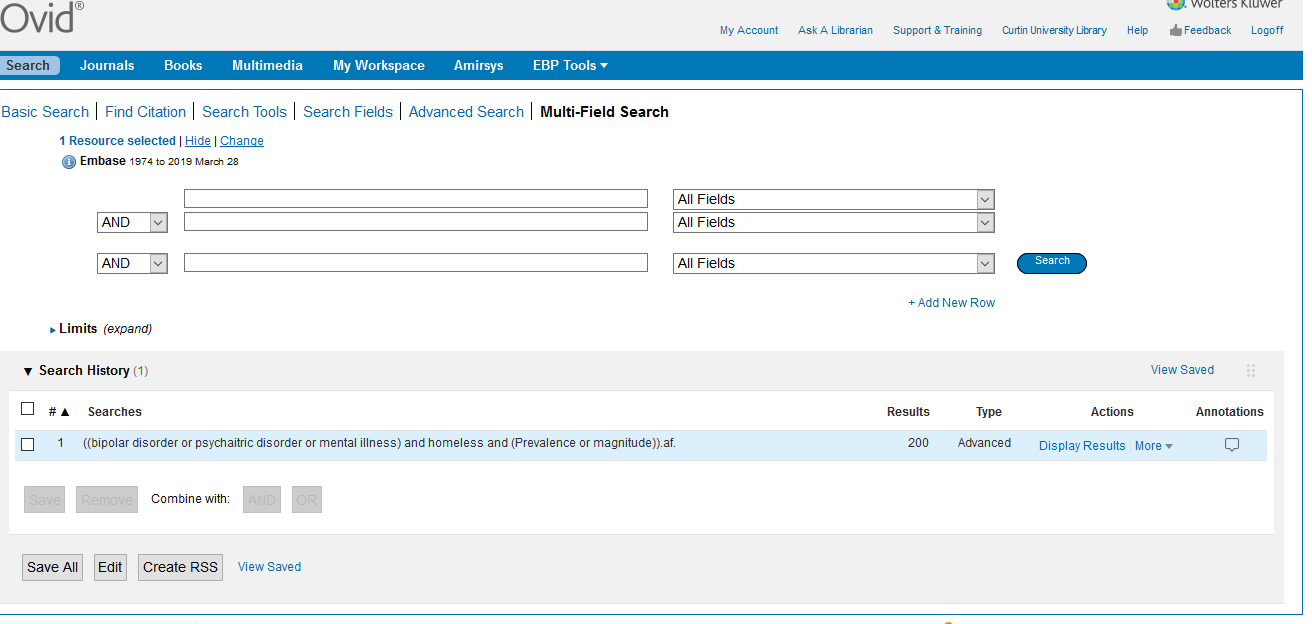


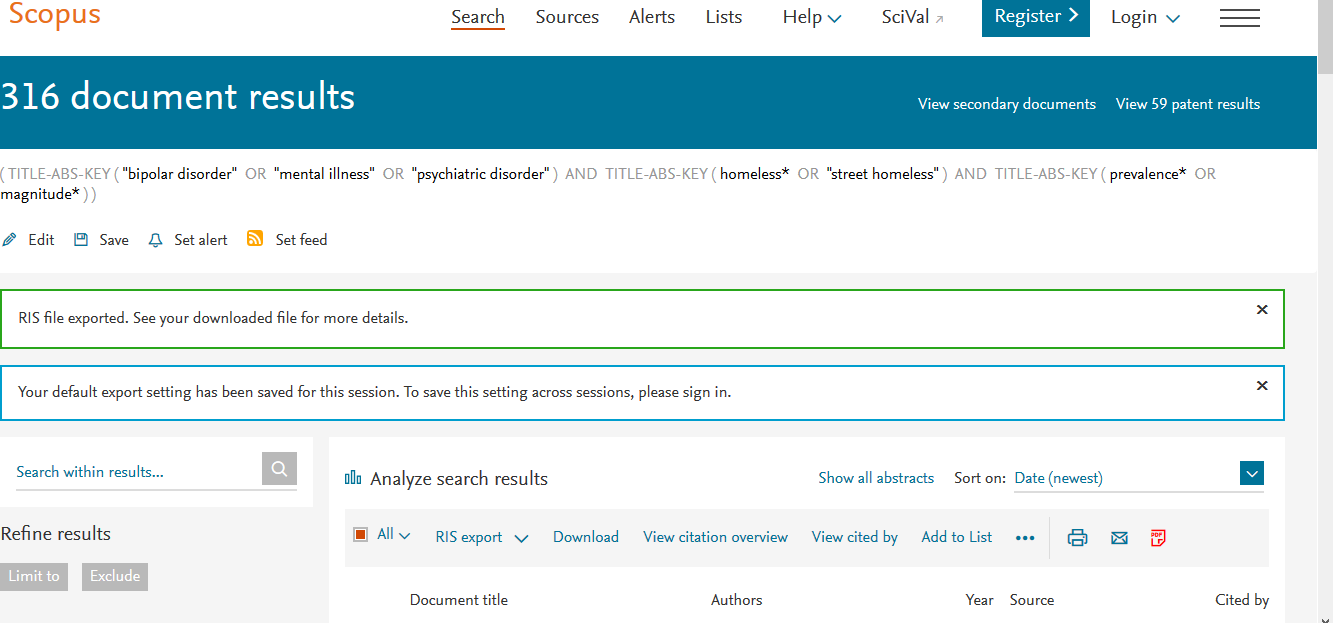

Supplement: Supplementary file 1 — Additional file 1: Screenshot of document results from the three databases. This additional material shows snapshot of number of studies identified during the database search PubMed (n = 2712), Embase (n = 200), and Scopus (n = 316). [file 12889_2020_8819_MOESM1_ESM.docx]
