# Supplementary material for: A systematic review and meta-analysis of the prevalence of bipolar disorder among homeless people
Source: BMC Public Health. 2020 Jun 9;20:731. doi: 10.1186/s12889-020-08819-x (PMC7282102; doi:10.1186/s12889-020-08819-x)
Supplement: Supplementary file 2 — Additional file 2: Excluded full text studies with reasons of exclusion. The major reasons for the exclusion includes: (a) Not measured the prevalence of bipolar disorder (n = 8): (b) the study population was not homeless people (n = 8); (2) reviews (n = 2); (3) duplicate (n = 2). [file 12889_2020_8819_MOESM2_ESM.docx]

**Supplementary file 3:** Excluded studies with the reasons for exclusion

The major reasons for the exclusion includes: (a) Not measured the prevalence of bipolar disorder (1-8); (b) the study population was not homeless people (9-16).. (c) Reviews (17, 18), and, (d) duplicate (19, 20).

1. Lauber C, Lay B, Rossler W. Homelessness among people with severe mental illness in Switzerland. Swiss medical weekly. 2005;135(3-4):50-6.

2. Yim LC-L, Leung HC-M, Chan WC, Lam MH-B, Lim VW-M. Prevalence of Mental Illness among Homeless People in Hong Kong. PloS one. 2015;10(10):e0140940.

3. Adams CE, Pantelis C, Duke PJ, Barnes TRE. Psychopathology, Social and Cognitive Functioning in a Hostel for Homeless Women. British Journal of Psychiatry. 1996;168(1):82-6.

4. Fekadu A, Hanlon C, Gebre-Eyesus E, Agedew M, Solomon H, Teferra S, et al. Burden of mental disorders and unmet needs among street homeless people in Addis Ababa, Ethiopia. BMC Medicine. 2014;12(1):138.

5. Ayano G, Assefa D, Haile K, Chaka A, Solomon H, Hagos P, et al. Mental, neurologic, and substance use (MNS) disorders among street homeless people in Ethiopia. Annals of general psychiatry. 2017;16(1):40.

6. Längle G, Egerter B, Albrecht F, Petrasch M, Buchkremer G. Prevalence of mental illness among homeless men in the community. Social Psychiatry and PsychiatricEpidemiology. 2005;40(5):382-90.

7. Bassuk EL, Rubin L, Lauriat A. Is homelessness a mental health problem? The American journal of psychiatry. 1984;141(12):1546-50.

8. Sarajlija M, Jugovic A, Zivaljevic D, Merdovic B, Sarajlija A. Assessment of health status and quality of life of homeless persons in Belgrade, Serbia. Vojnosanitetski pregled. 2014;71(2):167-74.

9. Bresnahan M, Collins PY, Susser E. Mental illness in an adult sample admitted to public hostels in the Rio de Janeiro metropolitan area, Brazil--on Lovisi et al. Social psychiatry and psychiatric epidemiology. 2003;38(9):499-501.

10. Okamura T, Takeshima T, Tachimori H, Takiwaki K, Matoba Y, Awata S. Characteristics of Individuals With Mental Illness in Tokyo Homeless Shelters. Psychiatric services (Washington, DC). 2015;66(12):1290-5.

11. Goodhew M, Salmon AM, Marel C, Mills KL, Jauncey M. Mental health among clients of the Sydney Medically Supervised Injecting Centre (MSIC). Harm reduction journal. 2016;13(1):29.

12. Greacen T, Finkelstein C. Mental health care in Paris. European psychiatry : the journal of the Association of European Psychiatrists. 2005;20 Suppl 2:S285-8.

13. Hamdullahpur K, Jacobs KWJ, Gill KJ. A comparison of socioeconomic status and mental health among inner-city Aboriginal and non-Aboriginal women. International journal of circumpolar health. 2017;76(1):1340693.

14. Kar SK, Sharma E, Agarwal V, Singh SK, Dalal PK, Singh A, et al. Prevalence and pattern of mental illnesses in Uttar Pradesh, India: Findings from the National Mental Health Survey 2015-16. Asian journal of psychiatry. 2018;38:45-52.

15. Kisely S, Chisholm P. Shared mental health care for a marginalized community in inner-city Canada. Australasian psychiatry : bulletin of Royal Australian and New Zealand College of Psychiatrists. 2009;17(2):130-3.

16. Albert M, McCaig LF. Emergency Department Visits Related to Schizophrenia Among Adults Aged 18-64: United States, 2009-2011. NCHS data brief. 2015(215):1-8.

17. Martens WH. A review of physical and mental health in homeless persons. Public health reviews. 2001;29(1):13-33.

18. Fazel S, Khosla V, Doll H, Geddes J. The prevalence of mental disorders among the homeless in western countries: systematic review and meta-regression analysis. PLoS medicine. 2008;5(12):e225.

19. Strehlau V, Torchalla I, Kathy L, Schuetz C, Krausz M. Mental health, concurrent disorders, and health care utilization in homeless women. Journal of psychiatric practice. 2012;18(5):349-60.

20. Kovess V, Mangin Lazarus C. The prevalence of psychiatric disorders and use of care by homeless people in Paris. Social psychiatry and psychiatric epidemiology. 1999;34(11):580-7.
