# Supplementary material for: A systematic review and meta-analysis of the prevalence of bipolar disorder among homeless people
Source: BMC Public Health. 2020 Jun 9;20:731. doi: 10.1186/s12889-020-08819-x (PMC7282102; doi:10.1186/s12889-020-08819-x)
Supplement: Supplementary file 3 — Additional file 3. The Quality of the included studies based on the NOS quality score, a 9 point score, Score 7 and above represented good quality; 6 to 7 represented moderate quality; and 0 and 5 represented poor quality. [file 12889_2020_8819_MOESM3_ESM.docx]

**Supplementary file 3:** The quality of studies included in final analysis

| Study name | Selection | Comparability | outcome | NOS score | Quality level |
| --- | --- | --- | --- | --- | --- |
| Koengel et.al. 1988 | 3 | 2 | 3 | 8 | High |
| Fichter, et. al. 2001 | 4 | 2 | 3 | 9 | High |
| Topolovec-Vranic et. al 2017 | 3 | 2 | 3 | 8 | High |
| Connolly et.al. 2008 | 2 | 1 | 2 | 5 | Low |
| Noel¨ et. al (2016) | 4 | 2 | 3 | 9 | High |
| Prinsloo. et.al 2012 | 2 | 1 | 1 | 4 | Low |
| Kovess et.al. 1999 | 4 | 2 | 3 | 9 | High |
| Greifenhagen et.al. 1997 | 2 | 1 | 2 | 5 | Low |
| Strehlua et.al. 2012 | 3 | 1 | 2 | 6 | Moderate |
| Heckert et.al. 1999 | 3 | 1 | 3 | 6 | Moderate |
